# Supplementary material for: Heat adaptation of phage T7 under an extended genetic code
Source: Virus Evol. 2021 Dec 1;7(2):veab100. doi: 10.1093/ve/veab100 (PMC8923235; doi:10.1093/ve/veab100)
Supplement: veab100_Supp [file veab100_supp.zip › Sequencing_Analysis.zip!/Sequencing_Analysis/A1/evidence/SNP_7.html]

BRESEQ :: Evidence


| Predicted mutation | | | | | | |
| --- | --- | --- | --- | --- | --- | --- |
| evidence | seq id | position | mutation | annotation | gene | description |
| RA | NC\_001604.1 | 26,753 | G→A | intergenic (–/–) | – / – | –/– |

| Read alignment evidence... | | | | | | | | | | | |
| --- | --- | --- | --- | --- | --- | --- | --- | --- | --- | --- | --- |
|  | seq id | position | | ref | new | freq | score (cons/poly) | reads | annotation | genes | product |
| \* | NC\_001604.1 | 26,753 | 0 | G | A | 99.1% | 6086.3 / NA | 2783 | intergenic (–/–) | *–/–* | –/– |
| Reads supporting (aligned to +/- strand):  **ref** base G (6/5);  **major** base A (1260/1475);  **minor** base C (24/2);  **total** (1298/1485) | | | | | | | | | | | |
| Fisher's exact test for biased strand distribution *p*-value = 1.02e-06 | | | | | | | | | | | |
| Kolmogorov-Smirnov test that lower quality scores support variant *p*-value = 1.00e+00 | | | | | | | | | | | |

Only 100 of 2789 total aligned reads displayed.  

`GTCGTGGTCTCATTGGGACTTTGGGGAAAACGTACAGGTTCTAGCTTGTCAGAGTATCAGCTCAGATATGTATGTGATTCTTCGCAATGAGTTCAATACGTTCCTAGCTAGAATCTCTTTCACTAAGAACGCCATTGACTTACAGGGAGAACCCTATCGTGCCTTTATGGACATGAAGATTCGATACACGATTCCTAGTGGAACATACAACGATGACACATTCACTACCTCTATTCATATTCCAACAATTTATGGTGCAAACTTCGGGAGGGGCAAAATCACTGTATTGGAGCCTGATGGTAAGATAACCGTGTTTGAGCAACCTACGGCTGGGTGGAATAGCGACCCTTGGCTGAGACTCAGCGGTAACTTGGAGGGACGCATGGTGTACATTGGGTTCAACATTAACTTCGTATATGAGTTCTCTAAGTTCCTCATCAAGCAGACTGCCGACGACGGGTCTACCTCCACGGAAGACATTGGGCGCTTACAGTTACGCCGAGCGTGGGTTAACTACGAGAACTCTGGTACGTTTGACATTTATGTTGAGAACCAATCGTCTAACTGGAAGTACACAATGGCTGGTGCCCGATTAGGC  >  NC_001604.1/26455‑27052`  

                                                                                                                                                                                                                                                                                                          `|`                                                                                                                                                                                                                                                                                                             
 `gagacagctcATTGGGACTTTGGTGAAAACGTACAGGTTCTAGCTTGTCAGAGTATCATCTCAGATATGTATTTGATTCTTCGCAATGAGTTCAATACGTTCCTAGCTAGAATCTCTTTCACTAAGAACGCCATTGACTTACAGTGAGAACCCTATCGTGCCTTTATGGACATGAAGATTCGATACACGATTCCTAGTGGAACATACAACGATGACACATTCACTACCTCTATTCATATTCCAACAATTTATGGTGCAAACTTCGGGAGGGGCAAAATCACTGTATTGGAGCCTGATAGTa                                                                                                                                                                                                                                                                                                          <  1:43405/294‑1 (MQ=255)`  
     `ggTCTCATTGGGACTTTGGGGAAAACGTACAGGTTCTAGCTTGTCAGAGTATCAGATCAGATATGTATGGGATTCTTCGCAATGAGTTCAATACGTTCCTAGCTAGAATCTCTTTCACTAATAACGCCATTGACTTACAGGGAGAACCCTATCGTGCCTTTATGGACATGAAGATTCGATACACGATTCCTAGTGGAACATACAACGATGACACATTCACTACCTCTATTCATATTCCAACAATTTATGGTGCAAACTTCGGGAGGGGCAAAATCACTGTATTGGAGCCTGATAGTAAGAt                                                                                                                                                                                                                                                                                                      <  1:358563/301‑1 (MQ=255)`  
      `gTCTCATTGGGACTTTGGGGAAAACGTACAGGTTCTAGCTTGTCAGAGTATCAGCTCAGATATGTATGTGATTCTTCGCAATGAGTTCAATACGTTCCTAGCTAGAATCTCTTTCACTAAGAACGCCATTGACTTACAGGGAGAACCCTATCGTGCCTTTATGGACATGAAGATTCGATACACGATTCCTAGTGGAACATACAACGATGACAAATTCACTACCTCTATTCATATTCCAACAATTTATGGTGCAAACTTCGGGAGTGGCAAAATCACTGTATTGGAGACTGATAGTAAGAtc                                                                                                                                                                                                                                                                                                     >  1:50537/1‑300 (MQ=255)`  
        `ttcATTGGGACTTTGGGGAAAACGTACAGGTTCTAGCTTGTCAGAGTATCAGCTCAGATATGTATGTGATTCTTCGCAATGAGTTCAATACGTTCCTAGCTAGAATCTCTTTCACTAAGAACGCCATTGACTTACAGGGAGAACCCTATCGTGCCTTTATGGACATGAAGATTCGATACACGATTCCTAGTGGAACATACAACGATGACACATTCACTACCTCTATTCATATTCCAACAATTTATGGTGCAAACTTCGGGAGGGGCAAAATCACTGTATTGGAGCCTGATAGTAAGATAAc                                                                                                                                                                                                                                                                                                   <  1:464068/300‑1 (MQ=255)`  
          `cATTGGGACTTTGGGGAAAACGTACAGGTTCTAGCTTGTCAGAGTATCAGCTCAGATATGTATGTGATTCTTCGCAATGAGTTAAATACGTTCCTAGCTAGAATCTCTTTCACTAAGAACGCCATTGACTTACAGGGAGAACCCTATCGTGCCTTTATGGACATGAAGATTCGATACACGATTCCTAGTGGAACATACAACGATGACACATTCACTACCTCTATTCATATTCCAACAATTTATGGTGCAAACTCCGGGAGGGGCAAAATCACTGTATTGGAGCCTGATCGTaatgtctctt                                                                                                                                                                                                                                                                                                 >  1:293243/1‑293 (MQ=255)`  
          `cATTGGGACTTTGGGGAAAACGTAAAGGTTCTAGCTTGACATAGTATAAGCGAAGATATGTATTGGATTCTTCTGAATGAGTTCAATACGTTCCTAGCTAGAATCTCTTTCACTAAGAACGACATTGACATACAGGGAGAACCCTATCGTGCCTGTATGGACATGAAGATTCGATACACGATTCCTAGTGGAACATACAACGATGACACATTCACTACCTCTATTCATATTCCAACAATTTATGGTGCAAACTTCGGGAGGGGAAAAATCACTGTATTGGAGCCTGATAGTAAGATAACCg                                                                                                                                                                                                                                                                                                 <  1:475633/301‑1 (MQ=255)`  
                `aaCTTTGTGGAAAACGTACAGGTTCTAGCTTGTCAGAGTATCAGCTCAGATATGTATGTGATTCTTCGCAATGAGTTCAATACGTTCCTAGCTAGAATCTCTTTCACTAAGAACGCCAATGACTTACAGGGAGAACCCTATCGTGCCTTTATGGACATGAAGATTCGATACACGATTCCTAGTGGAACATACAACGATGACACATTCACTACCTCTATTCATATTCCAACAATTTATGGTGCAAACTTCGGGAGGGGCAAAATCACTGTATTGGAGCCTGATAGTAAGATAACCGTGTTTg                                                                                                                                                                                                                                                                                           <  1:222918/300‑1 (MQ=255)`  
                             `aCGTACAGGTTCTAGCTTGTCAGAGTATCAGCTCAGATATGTATGTGATTCTTCGCAATGAGTTCAATACGTTCCTAGCTAGAATCTCTTTCACTAAGAACGCCATTGACTTACAGGGAGAACCCTATCGTGCTTTTATGGACATGAAGATTCGATACACGATTCCTAGTGGAACATACAACGATGACACATTCACTCCCTCTATTCCTCTTCCAACAATTTATGGTGCAACCTTCGGGAGGTGCCAAATCACTGTATTGGAGCCTGATAGTAAAATAACCGTGATTGAGCACACTACGGc                                                                                                                                                                                                                                                                              >  1:253320/1‑301 (MQ=255)`  
                             `aCGTACAGGTTCTAGCTTGTCAGAGTATCAGCTCAGATATGTATGTGATTCTTCGCAATGAGTTCAATACGTTCCTAGCTAGAATCTCTTTCACTAAGAACGCCATTGACTTACAGGGAGAACCCTATCGTGCCTTTATGGACATGAAGATTCGATACACGATTCCTAGTGGAACATACAACGATGACACATTCACTACCTCTATTCATATTCCAACAATTTATGGTGCAAACTTCGGGAGGGGCAAAATTACTGTATTGGAGCCTGATAGTAAGATAACCGTGTTTGAGCAACCTACGGc                                                                                                                                                                                                                                                                              <  1:445211/301‑1 (MQ=255)`  
                             `aCGTACAGGTTCTAGCTTGTCAGAGTATCAGCTCAGATATGTATGTGATTCTTCGCAATGAGTTCAATACGTTCCTAGCTAGAATCTCTTTCACTAAGAACGCCATTGACTTACAGGGAGAACCCTATCGTGCCTTTATGGACATGAAGATTCGATACACGATTCCTAGTGGAACATACAACGATGACACATTCACTACCTCTATTCATATTCCAACAATTTATGGTGCAAACTTCGGGAGGGGCAAAATCACTGTATTGGAGCCTGATAGTAAGATAACCGTGTTTGAGAAACCTACGGc                                                                                                                                                                                                                                                                              >  1:424054/1‑301 (MQ=255)`  
                                    `ggagatgtgtataagagacagccgCTCAGATATGTATGTGATTCTTCGCAATGATTTCAATACGTTCCTAGCTAGAATCTCTTTCACTAAGAACGCCATTGACTTACAGGGAGAACCCTATCGTGCCCTTATGGACATGAAGATTCGATACACGATTCCTAGTGGAACATAAAACGAGGACACATTCACTACCTCTATTCATATTCCAACAATTTATGGTGCAAACTTCGGGAGGGGCAAAATCACTGTATTGGAGCCTGATAGTAAGATAACCGTGTTTGAGCAACCTACGGCTGGGTgg                                                                                                                                                                                                                                                                       <  1:313848/278‑1 (MQ=255)`  
                                    `ggTTCTAGCTTGTCAGAGTATCAGCTCAGATATGTATGTGATTCTTCGCAATGAGTTCAATACGTTCCTAGCTAGAATCTCTTTCACTAAGAACGCCATTGACTTACAGGTAGAACCCTATCGTGCCTTTATGGACATGAAGATTCGATACACGATTCCTAGTGGAACATACAACGATGCCACATTCACTCCCTCTATTCATATTCCAACAATTTATGGTGCAAACTTCGGGAGGGGCAAAATCACTGTCTTGACGCCTGATAGTAAGATAACCGTGTTTGAGCACCCTACGTCTGGGTgg                                                                                                                                                                                                                                                                       >  1:233037/1‑301 (MQ=255)`  
                                         `tAGCTTGTCAGGGTCTCAGCTCAGATATGTATGTGATTCTACGCACTGAGTTAAATACATTCATATCAAGAATCTCTTGAAATAAGAAGTAAATTGAATTACAGGGAGAACACTATCGTGCCTGTATGGACATGAAGATAAGATACACGATTCATAGTGGAAAATACAACGATGAAACATTAAATACCTCTATGCATATTCCAACAAAGTATGGTGCAAACTTCGGGAGGGGCAAAATCACTGTATTGGAGCCTGATAATAAGATAACCGTGTTTGAGCAACCTACGGCTGAGTGGAATAg                                                                                                                                                                                                                                                                  <  1:245508/301‑1 (MQ=255)`  
                                          `aGCTTGTCAGAGTATCAGCTCAGATATGTATGTGATTCTTCGCAATGAGTTCAATACGTTCCTAGCTAGAATCTCTTTCACTAAGAACGCCATTGACTTCCAGGGAGAACCCTATCGTGCCTTTATGGACATGAAGATTCGATACACGATTCCTAGTGGAACATACAACGATGACACATTCACTACCTCTATTCATATTCCAACAATTTATGGTGCAAACTTCGGGAGGGGCAAAATCACTGTATTGGAGCCTGATAGTAAGATAACCGTGTTTGAGCAACCTACGGCTGGGTGGAATAGc                                                                                                                                                                                                                                                                 >  1:206950/1‑301 (MQ=255)`  
                                                 `cAGAGTATCAGCTCAGATATGTATGTGATTCTTCGCAATGAGTTCAATACGTTCCTAGCTAGAATCTCTTTCACTAAGAACGCCATTGACTTACAGGGAGAACCCTATCGTGCCTTTATGGACATGAAGATTCGATACACGATTCCTAGTGGAACATACAACGATGACACATTCACTACCTCTATTCATATTCCAACAATTTATGGTGCAAACTTCGGGTGGGGCAAAATCACTGTATTGGAGCCTGATAGTAAGATAACCGTGTTTGATCAACCTACGGCTGGGTGGAATACCCACCCtt                                                                                                                                                                                                                                                          >  1:300082/1‑301 (MQ=255)`  
                                                   `gagTATCAGCTCAGATATGTATGTGATTCTTCTCAATGAGTTCAATACGTTCCTAGCTCGAATCTCTTTCACTAAGAACGCCATTGACTTACAGGGAGAACCCTATCGTGCCTTTATGGACATGAAGATTCGATACACGATTCCTAGTGGAACATACAACGATGACACATTCACTACCTCTATTCATATTCCAACAATTTATCGTGCAAACTTCGGGCGGGGCAAAATCACTGCATTGTAGCCTGCTAGTAAGATAACCGTGTTTGAGCAACCTACGCCTGGTTGTACTAGctgtctctta                                                                                                                                                                                                                                                        >  1:104473/1‑292 (MQ=255)`  
                                                     `gTATCAGCTCAGATATGTATGTGATTCTTCGCAATGAGTGCAATACGTTCCTAGCTAGAATCTCTTTCACTAAGAACGCCATTGACTTACAGGGAGAACCCTATCGTGCCTTTATGGACATGAAGATTCGATACACGATTCCTAGTGGAACATACAACGATGACACATTCACTACCTCTATTCATATTCCAACAATTTATGGTGCAAACTTCGGGAGGGGCAAAATCACTGTATTGGAGCCTGATCGTAAGATAACCGTGTTTGAGCAACCTACGGCTGGGTGGAATAACGACCCTTGGCt                                                                                                                                                                                                                                                      >  1:52526/1‑301 (MQ=255)`  
                                                           `gCTCAGATATGTATGTGATTCTTCGCAATGAGTTCAATACGTTCCTAGCTAGAATCTCTTTCACTAAGAACGACATTGACTTACAGGGAGACCCCTATCGTGCCTTTATGGACATGAAGATTCGATACACGATTCCTAGTGGAACATACAACGATGACACATTCACTACCTCTATTCATATTCCAACAATTTATGGTGCACACTTCGGGAGGGGCAAAATCACCGTATTGGAGCCTGATAGTAAGATAAACGTGTTTGAGCAACCTACGGCTGGGTGGAATAGCGACCCTTGGCGGGGAcc                                                                                                                                                                                                                                                >  1:321671/1‑300 (MQ=255)`  
                                                                `gATATGTATGTGATTCTTCGCAATGAGTTCAATACGTTCCTAGCTAGAATATCTTTCACTAAGAACGCCATTGACTTACAGGGAGAACCCTATTGTGCCTTTATGGACATGAAGATTCGATACACGATTCCTAGTGGAACATACAACGATGACACATTCACTACCTCTATTCATATTCCAACAATTTATGGTGCAAACTTCGGGAGGGGCAAAATCACTGTATTGGAGCCTGATAGTAAGATAACCGTGTTTGAGCAACCTACGGCTGGGTGGAATAGCGACCCTTGGCTGAGACTCAGCg                                                                                                                                                                                                                                           >  1:24160/1‑301 (MQ=255)`  
                                                                 `atatGTATGTGATTCTTCGCAATGAGTTCAATACGTTCCTAGCTAGAATCTCTTTCACTAAGAACGCCATTGACTTACAGGGAGAACCCTATCGTGCCTTTATGGACATGAAGATTCGATACACGATTCCTAGTGGAACATACAACGATGACACATTCACTACCTCTATTCATATTCCAACAATTTATGGTGCAAACTTCGGGAGGGGCAAAATCACTGTATTGGAGCCTGATAGTAAGATAACCGTGTTTGAGCAACCTACGGCTGGGTGGAATAGCGACCCTTGGCTGAGACTCAGCgg                                                                                                                                                                                                                                          >  1:471874/1‑301 (MQ=255)`  
                                                                 `atatGTATGTGATTCTTCGCAATGAGTTCAATACGTTCCTAGCTAGAATCTCTTTCACTAACAACGCCATTGACTTACAGGGAGAACCCTATCGTGCCTTTATGGACATGAAGATTCGATACACGATTCCTAGTGGAACATACAACGATGACACATTCACTACATCTATTCATATTCTAACAATTTATGGTGCAAACTTCGGGAGGGGCACAATCACTGTATTGGCGCCTGATAGTAAGATAACCGTGTTTGAGCAACCTACGGCTGGGTGGCATAGCGACCCTTGGCTGCGACTCATCgg                                                                                                                                                                                                                                          >  1:167665/1‑301 (MQ=255)`  
                                                                     `ggagatgtgtataagagacaggTTAAATACGTTCCTAGCTAGAAGCTCTTTCACTAAGAACGCCATTGACTTACAGGGAGAATCCTATCGTGCCTTTATGGACATGAAGATTCGATACACGATTCCTAGTGGAACATACCACGATGACACATTCACTACCTCTATTCATATTCCAACAATTTATGGTGCAAACTTCGGGAGGGGCAAAATCACTGTATTGGAGCCTGATAGTAAGATAACCGTGTTTGAGCAACCTACGGCTGGGTGGAATAGCGACCCTTGGCTGAGACTCAGCGGTAAc                                                                                                                                                                                                                                      <  1:287109/280‑1 (MQ=255)`  
                                                                      `aatgtGATTCTTCGAAATGAGTTCAATACGTTCCTAGCTAGAATCTCTTTCACTAAGAACGCCATTGACTTACAGGGAGAACCCTATCGTGCCTTTATGGACATGAAGATTCGATACACGATTCCTAGTGGAACATACAACGATGACACATTCACTACCTCTATTCATATTCCAACAATTTATGGTGCAAACTTCGGGAGGGGCAAAATCACTGTATTGGAGCCTGATAGTAAGATAACCGTGTTTGAGCAACCTACGGCTGGGTGGAATAGCGACCCTTGGCTGAGACTCAGCGGTAACt                                                                                                                                                                                                                                     <  1:39205/300‑1 (MQ=255)`  
                                                                       `atgtGATTCTTCGCAATGAGTTCAATACGTTCCTAGCTAGAATCTCTTTCACTAAGAACGCCATTGACTTACAGGGAGAACACTATCGTGCCTTTATGGACATGAAGATTCGATACACGATTCCTAGTGGAACATACAACGATGACACATTCACTACCTCTATTCATATTCCAACAATTTATGGTGCAAACTTCGGGAGGGGCAAAATCACTGTATTGGAGCCTGATAGTAAGATAACCGTGTTTGAGCAACCTACGGCTGGGTGGAATAGCGACCCTTGGCTGAGACTCAGCGGTAACtt                                                                                                                                                                                                                                    >  1:428868/1‑301 (MQ=255)`  
                                                                           `aaTTCTTCGCAATGAGTTTAAGACGTTACTAGGTAGAATCTCTTTCAATAAGAACGCCATTGACTTACAGGGAGAACCCTATCGTGCCTTTATGGACATGAAGATTCGATACACGATTCCTAGTGGAACATACAACGATGACACATTCACTACCTCTATTCATATTCCAACTATTTATGGTGCAAACTTCGGGAGGGGCAAAATCACTTTATTGGAGCCTGATAGTAAGATAACCGTGTTTGAGCAACCTACGGCTGGGTGGAATAGCGACCCTTGGCTGAGACTCAGCTGTAACTTGGAg                                                                                                                                                                                                                                <  1:296533/300‑1 (MQ=255)`  
                                                                            `aTTCTTCGCAATGAGTTCAATACGTTCCTAGCTAGAATCTCTTTCACTAAGAACGCCATTGACTTACAGGGAGAACCCTATCGTGCCTTTATGGACATGAAGATTCGATACACGATTCCTAGTGGAACATACAACGATGACACATTCACTACCTCTATTCATATTCCAACAATTTATGGTGCAAACTTCGGGAGGGGCAAAATCACTGTATTGGAGCCTAGTAGTACATTAACCGTGTTGGAGCAACCTAGGGCTGGTTGGAATAGCGACCCTTGGCTgcagctcggcgcgagccctaaaa                                                                                                                                                                                                                               >  1:376530/1‑279 (MQ=255)`  
                                                                                   `gagacagtttCAATGCGTTCCTAGCTCGAAACTCTTTCACTAAGAACGCCATTGACTTACAGGAAGAACCCTATCGTGCCTTTATGGACATGAAGATTCGATACACGATTCCTAGTGGAACATACAACGATGACACATTCACTACCTCTATTCATATTCCATCAATTTATGGTGCAAACTTCGGGAGGGGCAAAATCACTGTATTGGAGCCTGATAGTAAGATAACCGTGTTTGAGCAACCTACGGCTGGGTGGAATAGCGACCCTTGGCTGCGACTCAGCGGTAACTTGGAGGGACGCAt                                                                                                                                                                                                                        <  1:352892/293‑1 (MQ=255)`  
                                                                                    `cAATGAGTTCAATACGTTCCTAGCTAGAATCTCTTTCACTAAGAACGCCATTGACTTACAGGGAGAACCCTATCGTGCCTTTATGGACATGAAGATTCGATACACGATTCCTAGTGGAACATACAACGATGACACATTCACTACCTCTATTCATATTCCAACAATTTATGGTGCAAACTACGGGAGGGGCAAAATCACTGTATTGACGCATGATAGAAAGATAACCGTGTATGAGCAACCTACGGCTGGGTGGAATAGCGACCCATGGCTTGGACTCAGCGGTAAATAGGAGGTACGAATg                                                                                                                                                                                                                       >  1:450116/1‑301 (MQ=255)`  
                                                                                        `ctcggagatgtgtataagagacagaTCTCTTTCACTAAGAACGCCATTGACTTACAGGGAGAACCCTATCGTGCCTTTATGGACATGAAGATTCGATACACGATTCCTAGTGGAACATACAACGATGACACATTCACTACCTCTATTCATATTCCAACAATTTATGGTGCAAACTTCGGGAGGGGCAAAATCACTGTATTGGAGCCTGATAGTAAGATAACCGTGTTTGAGCAACCTACGGCTGGGTGGAATAGCGACCCTTGGCTGAGACTCAGCGGTAACTTGGAGGGACGCATGgtgt                                                                                                                                                                                                                   <  1:108088/277‑1 (MQ=255)`  
                                                                                          `gTTCAATACGTTCCTAGCTAGAATCTCTTTCACTAAGAACGCCATTGACTTACAGGGAGAACCCTATCGTGCCTTTATGGACATGAAGATTCGATACACGATTCCTAGTGGAACATACAACGAAGACACATTCACTACCTCTATTCATATTCCAACAATTTATGGTGCAACCTTCGGGAGGGGCAAAATCACTGTATTGGAGCCTGTTAGTAAGATAACCGTGTTTGAGCAACCTACGGCTGGGTGGAATCGCGACCCTTTGCTGAGACTCAGCGGTAACTTGGAGGGACGCATGGTGTaa                                                                                                                                                                                                                 >  1:348508/1‑300 (MQ=255)`  
                                                                                             `gagacagtgccTAGCTAGAATATTTTTAAATAAGAACGGCATTGACTTAAAGGGAGAACCCTATCGTGCCTTTATGGACATGAAGATTCGATAAACGATTCCTAGTGGAACATACAACGATGACACATTCACTAACTCTATTCATATTCCAACAATTTATGGTGCAAACTTCGGGAGGGGCAAAATCACTGTATTGGAGCCTGATAGTAAGATAACCGTGTTTGAGCAACCTACGGCTGGGTGGAATAGCGACCCTTGGCTGAGACTCAGCGGTAACTTGGAGGGACGCATGGTGTACAtt                                                                                                                                                                                                              <  1:485355/292‑1 (MQ=255)`  
                                                                                             `cAATACGTTCCTAGCTATAATCTCTTTCACTAATCACGCCATTGACTTACAGGGATAACCCTATCGTGCCTTTATGGACATGAAGATTCGATACACGATTCCTAGTGGAACATACAACTATGACACATTCACTACCTCTATTCATATTCCAACAATTTATGGTGCAAACTTCGGGATGGGCAAAATCACTGTATTGGAGCCTGATAGTAAGATAACCGTGTTTGAGCAACCTACGGCTGGGTGGAATAGCGACCCTTGGCTGAGACTCAGCGGTAACTTGGAGGGACGCATGGTGTACAtt                                                                                                                                                                                                              <  1:281435/301‑1 (MQ=255)`  
                                                                                              `aaTACGTTCCTAGCTAGAATCTCTTTCACTAAGAACGCCATTGACTTACAGGGAGAACCCTATCGTGCCTTTATGGACATGAAGATTCGATACACGATTCCTAGTGGAACATACAACGATGACACATTCACTACCTCTATTCATATTCCAACAATTTATGGTGCAAACTTCGGGAGGGGCAAAATCACTGTATTGGAGCCTGATAGTAAGATAACCGTGTTTGAGCAACCTACGGCTGGGTGGAATAGCGACCCTTGGCTGAGACTCAGCGGTAACTTGGAGGGACGCATGGTGTACATTg                                                                                                                                                                                                             <  1:453913/301‑1 (MQ=255)`  
                                                                                                 `aCGTTCCTAGCTAGAATTTCTTTCACTAAGAACGCCATTGACTTAAAGGGAGAACCCTATCGTGCCTTTATGGACATGAAGATTCGATACACGAGTCCTAGTAGAACATACAACGATGACACATTCACTACCTCTATTCATATTCCAACAATTTATGGTGCAAACTTCGGGAGGGGCAAAATCACTGTATTGGAGCCTGATAGTAAGATAACCGTGTTTGAGCAACCTACGGCTGGGTGGAATAGCGACCCTTGGCTGAGACTCAGCGGTAACTTGGAGGGACGCATGGTGTACATTGGGt                                                                                                                                                                                                          <  1:82444/301‑1 (MQ=255)`  
                                                                                                    `ttCCTAGCTAGAATCTCTTTCACTAAGAACGCCATTGACTTACAGGGAGAACCCTATCGTGCCTTTATGGACATGAAGATTCGATACACGATTCCTAGTGGAACATACAACGATAACACATTCACTACCTCTATTCATATTTCAACAAACTATCGTGCAAACTTCGGGAGGGGCAAAATCCCTTTATTGCAGCCTGATAGTAAGATCACCGTGTTTGAGCAACCTACGGCTGGATAGATTAGCGACCCTTTGCTGAGACTCAGCGGTGACTTGGCGGGACGCATCGTGTACATTggatcct                                                                                                                                                                                                       >  1:318154/1‑296 (MQ=255)`  
                                                                                                            `tagAATCTCTTTCACTAAGAACGCCATTGACTTACAGGGAGAACCCTATCGTGCCTTTATGGACATGAAGATTCGATACACGATTCCTAGTGGAACATACAACGATGACACATTCACTACCTCTATTCATATTCCAACAATTTATGGTGCAAACTTCGGGAGGGGCAAAATCACTGTATTGGAGCCTGATAGTAAGATAACCGTGTTTGAGCAACCTACGGCTGGGTGGAATAGCGACCCTTGGCTGAGACTCAGCGGTAACTTGGAGGGACGCATGGTGTACATTGGGTTCAACATTAAc                                                                                                                                                                                               <  1:180689/301‑1 (MQ=255)`  
                                                                                                             `tgAATCTCTTTCACTAAGAACGCCATTGACTTACAGGGAGAACCCTATCGTGCCTTTATGGACATGAAGATTCGATACACGATTCCTAGTGGAACATACAACGATGACACATTCACTACCTCTATTCATATTCCAACAATTTATGGTGCAAACTTCGGGAGGGGCAAAATCACTGTATTGGAGCCTGATAGTAAGATAACCGTGTTTGAGCAACCTACGGCTGGGTGGAATAGCGATCCTTGGCTGAGACTCAGCGGTAACTTGGATGGACGCATGGTGTACATTGGGTTCAACATTAACt                                                                                                                                                                                              <  1:111863/300‑1 (MQ=255)`  
                                                                                                             `agAAACTCTATCACTAAGAACACCATTGACTTACAGGGAGAACGCTATCGTGCCTTTATGGACATGAAGATTCGATACACGATTCATAGTGGAACATACAAAGATGACACATTCACTAACTCTATACATATTCCAACTATTTATGGTGCAAACGTCGGGAGGGGCAAAAGCACTGTAATGGAGCCTGATAGTAAGATAACCGTGTTTGAGCAACCTACGGCTGGGTGGAATAGCGACCCTTGGCTGAGACTCAGCGGTAACGTGGAGGGACGCATGGTGTACATTGGGTTCAACATTAACt                                                                                                                                                                                              <  1:499074/301‑1 (MQ=255)`  
                                                                                                              `aaaTCTCTTTCACTAAGAACGCCATTGACTTACAGGGAGAACCCTATCGTGCCTTTATGGACATGAAGATTCGATACACAATTCCTAGTGGAACATACAACGATGACACATTAACTACCTCTATTCATATTCCAACAATTTATGGTGCAAACTTCGGGAGGGGCAAAATCACTGTATTGGAGCCTGATAGTAAGATAACCGTGTTTGAGCAACCTACGGCTGGGTGGAATAGCGACCCTTGGCTGAGACTCAGCGGTAACTTGGAGGGACGCATGGTGTACATTGGGTTCAACATTAACtt                                                                                                                                                                                             <  1:3715/300‑1 (MQ=255)`  
                                                                                                                    `gtgtataagagacagacATGGACTTACAGGGAGAACCCTATCGTTCCTTTATGGACATGAAGATTCGATACACGATTCCTAGTGGAACATACAACGATGACACATTCACTACCTCTATTCATATTCCAACAATTTATGGTGCAAACTTCGGGAGGGGCAAAATCACTGTATTGGAGCCTGATAGTAAGATAACCGTGTTTGAGCAACCTACGGCTGGGTGGAATAGCGACCCTTGGCTGAGACTCAGCGGTAACTTGGAGGGACGCATGGTGTACATTGGGTTCAACATTAACTTCGtata                                                                                                                                                                                       <  1:307745/285‑1 (MQ=255)`  
                                                                                                                    `gagatttgtataggagacaggACTTACAGGGAGAACCCTATCGTTCCTTTATTGACATTAAGATTCTATACACGATTCCTAGTGGAACATACAACGATGACACATTCACTACCTCTATTCATCTTCCAACAATTTATGGTGCAAACTTCAGGAGGGGCAAATTCCCTTTATTGGAGCCTGATAGTAAGATAACCGTGTTTGAGCAACCTACGGCTGGGTGGAATAACGACCCCTGGCTTAGACTCAGCGGTAACTTGGAGGGACGCATGGTGTACATTGGGTTCAACATTAACTTCGtata                                                                                                                                                                                       <  1:325223/281‑1 (MQ=255)`  
                                                                                                                        `atgtgtataagggacagaattACAGGGAGAACCCTATCGTGCCTTTATGGACATGAAGATTCGATACACGATTACTAGTGGAACATACAACGATGACACATTCACTACCTCTATTCATATTCCAACAATTTATGGTGCAAACTTCGGGAGGGGCAAAATCACTGTATTGGAGCCTGATAGTAAGATAACCGTGTTTGGGCAACCTACGGCTGGGTGGAATAGCGACCCTTGGCTGAGACTCAGCGGTAACTTGGAGGGACGCATGGTGTACATTGGGTTCAACATTAACTTCGTATATGAg                                                                                                                                                                                   <  1:494243/282‑1 (MQ=255)`  
                                                                                                                        `aatgtggataagagacagcTTACAGGTATAACCCTATCGTGCCTTTATGGACATGAAGATTCGATACAACATTCCTAGTGGAACATACAACGATGACAAATTCACTCCCTCTATTCATATTCCAACAAATTATGGTGCAAACTTCGGGAGTGGCAAAATCACTGTATTTGAGCCAGATAGTAAGATAACCGTGTTTGAGCAACCTACGGCTGGGTGGAATAGCGACCCTTGGCTGAGACTCAGCGGTAACTTGGAGGGACGCATGGTGTACATTGGGTTCAACATTAACTTCGTATATGAg                                                                                                                                                                                   <  1:249541/283‑1 (MQ=255)`  
                                                                                                                        `aaCTAAGAACGCCATTGACTTACAGGGAGAACCCTATCGTGCCTTTATGGACATGAAGATTCGATACACGATTCCTAGTGGAACATACAACGATGACACATTCACTACCTCTATTCATATTCCAACAATTTATGGTGCAAACTTCGGGAGGGGCAAAATCACTGTATTGGAGCCTGATAGTAAGATAACCGTGTTTGAGCAACCTACGGCTGGGTGGAATAGCGACCCTTGGCTGAGACTCAGCGGTAACTTGGAGGGACGCATGGTGTACATTGGGTTCAACATTAACTTCGTATATGAg                                                                                                                                                                                   <  1:100097/300‑1 (MQ=255)`  
                                                                                                                                 `cGCCATTGACTTACAGGGAGAACCCTATCGTGCCTTTATGGACATGAAGATTCGATACACGATTACTAGTGGAACATACAACGATGACACATTCACTACCTCTATTCATATTCAAACAATTTATGGTGCAAACTTCGGGAGGGGCAAAATCACTGTATTGGAGCCTGATAGTAAGATAACCGTGTTTGAGCAACCTACGGCTGGGTGGAATAGCGACCCTTGGCTGAGACTCAGCGGTAACTTGGAGGGACGCATGGTGTACATTGGGTTCAACATTAACTTCGTATATGAGTTCTCTAAg                                                                                                                                                                          <  1:198735/301‑1 (MQ=255)`  
                                                                                                                                  `gCCATTGACTTACAGGGAGAACCCTATCGTGCCTTTATGGACATGAAGATTCGATACACGATTCCTAGTGGAACATACAACGATGACACATTCACTACCTCTATTCATATTCCAACAATTTATGGTGCAAACTTCGGGAGGGGCAAAATCACTGTATTGGAGCCTGATAGTAAGATAACCGTGTTTGAGCAACCTACGGCTGGGTGGAATAGCGACCCTTGGCTGAGACTCAGCGGTAACTTGGAGGGACGCATGGTGTACATTGGGTTCAACATTAACTTCGTATATGAGTTCTCTAAGt                                                                                                                                                                         <  1:127888/301‑1 (MQ=255)`  
                                                                                                                                   `ccATTGACTTACAGGGAGAACCCTATCGTGCCTTTATGGACATGAAGATTCGATACACGATTCCTAGTGGAACATACAACGATGACACATTCACTACCTCTATTCATATTCCAACAATTTATGGTGCAAACTTTGGGAGGGGCAAAATCACTGTATTGGAGCCTGATAGTAAGATAACCGTGTTTGAGCAACCTACGGCTGGGTGGAATAGCGACCCTTGGCTGAGACTCAGCGGTAACTTGGCGGGACGCATGGGGTACATTGGGTACCACAGTGACTTctttctcttataacaatctcc                                                                                                                                                                        >  1:91987/1‑281 (MQ=255)`  
                                                                                                                                         `aCTTACAGGGAGAACCCGATCGTGCCTTTATGGACATGAAGATTCGATACACGATTCCTAGTGGAACATACAACGATGACACATTCACTACCTCTATTCATATTCCAACAATTTATGGTGCAAACTTCGGGAGGGGCAAAATCACTGTATTGGAGCCTGATAGTAAGATAACCGTGTTTGAGCAACCTACGGCTGGGTGGAATAGCGACCCTTGGCTGAGACTCAGCGGTAACTTGGAGGGACGCATGGTGTACATTGGGTTCAACATTAACTTCGTATATGAGTTATCTAAGTTCCtcat                                                                                                                                                                  <  1:56156/301‑1 (MQ=255)`  
                                                                                                                                             `aCAGGGAGAACCCTATCGTGCCTTTATGGACATGAAGATTCGATACACGATTCCTAGTGGAACATACAACGATGACACATTCACTACCTCTATTCATATTCCAACAATTTATGGTGCAAACTTCGGGAGGGGCAAAATCACTGTATTGGAGCCTGATAGTAAGATAACCGTGTTTGAACAACCTACGGCTGGGTGGAATAGCGACCCTTGGCTGAGACTCAGCGGTAACTTGGAGGGACGCATGGTGTACATTGGGTTCAACATTAACTTCGTATATGAGTTCTCTAATTTCCTCATAAAg                                                                                                                                                              >  1:304003/1‑301 (MQ=255)`  
                                                                                                                                              `cAGGGAGACCCCTATCGTGCCTTTATGGACATGAAGATTCGATACACGATCCCTAGTGGAACATACAACGATGACACATTCACTACCTCTATTCATATTCCAACAATTTATGGTGCAAACTTCGGGAGGGGCAAAATCACTGTATTGGAGCCTGATAGTAAGATAACCGTGTTTGAGCAACCTACGGCTGGGTGGAATAGCGACCCTTGGCTGAGACTCAGCGGTAACTTGGAGGGACGCATGGTGTACATTGGGTTCAACATTAACTTCGTATATGAGTTCTCTAAGTTCCTCATCAAGc                                                                                                                                                             <  1:468618/301‑1 (MQ=255)`  
                                                                                                                                              `aaGGGAGAACCCTATCGTGCCTTTATGGACATGAAGATTCGATACACGATTCCTAGTGGAACATACAACGATGACACATTCACTACCTCTATTCATATTCCAACAATTTATGGTGCAAACTTCGGGAGGGGCAAAATCACTGTATTGGATCCTGATAGTAAGATAACCGTGTTTGAGCAACCTACGGCTGGGTGGAATAGCGACCCTTGGCTGAGACTCAGCGGTAACTTGGAGGGACGCATGGTGTACATTGGGTTCAACATTAACTTCGTATATGAGTTCTCTAAGTTCCTCATCAAGc                                                                                                                                                             <  1:162863/300‑1 (MQ=255)`  
                                                                                                                                                `gggAGAACCCTATCGTGCCTTTATGGACATGAAGATTCGATACACGATTCCTAGTGGAACATACAACGATGACACATTCACTACCTCTATTCATATTCCAACAATTTATGGTGCAAACTTCGGGAGGGGCAAAATCACTGTATTGGAGCCTGATAGTAAGATAACCGTGTTTGAGCAACCTACGGCTGGGTGGAATAGCGACCCTTGGCTGAGACTCAGCGGTAACTTGGAGGGACGCATGGTGTACATTGGGTTCAACATTAACTTCGTATATGAGTTCTCTAAGGTCCTCATCAAGCaa                                                                                                                                                           >  1:257824/1‑300 (MQ=255)`  
                                                                                                                                                       `cccTATCGTGCATTTATGGACATGAAGATTCGATACACGATTCCTAGTGGAACATACAACGATGACACATTCACTACCTCTATTCATATTCCAACAATTTATGGTGCAAACTTCGGGAGGGGCAAAATCACTGTATTAGAGCCTGATAGTAAGATAACCGTGTTTGAGCAACCTACGGCTGGGTGGAATAGCGACCCTTGGCTGAGACTCAGCGGTAACTTGGAGGGGCGCATGGTGTACATTGGGTTCAACATTAACTTCGTATATGAGTTCTCTAAGTTCCTCATCAAGCAGACTGCcg                                                                                                                                                    >  1:236136/1‑301 (MQ=255)`  
                                                                                                                                                         `cTATCGTGCCTTTATGGACATGAAGATTCGATACACGATTCCTAGTGGAACATACAACGATGACACATTCACTACCTCTATTCATATTCCAACAATTTATGGTGCAAACTTCGGGAGGGGCAAAATCACTGTATTGGAGCCTTATAGTAAGATAACCGTGTTTGAGCAACCTACGGCTGGGTGGAATAGCGACCCTTGGCTGAGACTCAGCGGTAACTTGGAGGGACGCATGGTGTACATTGGGTTCAACATTAACTTCGTATATGAGTTCTCTACGTTCCtcctgtctcttatacacatt                                                                                                                                                  >  1:13606/1‑283 (MQ=255)`  
                                                                                                                                                          `tATCGTGCCTTTATGGACATGAAGATTCGATACACGATTCCTAGTGTAACATACAACGATGACACATTCACTACCTCTATTCATATTCCAACAATTTATGGTGCAAACTTCGGGAGGGGCAACATCACTGTATTGGAGCCTGATAGTAAGATAACCGTGTTTGAGCAACCTACGGCTGGGTGGAATAGCGACCCTTGGCTGAGACTCAGCGGTAACTTGGAGGGCCGCATGGTGTACATTGGGTTCCACATTAACTTAGTATATGAGTTCTCTAAGTTCCTCATCCAGcctgtctcttatt                                                                                                                                                 >  1:227172/1‑289 (MQ=255)`  
                                                                                                                                                            `gtgtataagagacagcATGAAGATTCGATACACGATTCCTAGTGGAACATACTACGATGACACATTCACTACCTCTATTCATATTCCAACAATTTATGGTGCAAACTTCTGGAGGGGCAAAATCACTGTATTGGAGCCTGATAGTAAGATAACCGTGTTTGAGCAACCTACGGCTGGGTGGAATAGCGACCCTTGGCTGAGACTCAGCGGTAACTTGGAGGGACGCATGGTGTACATTGGGTTCAACATTAACTTCGTATATGAGTTCTCTAAGTTCCTCATCAAGCAGACTGCCgacgac                                                                                                                                               <  1:202509/286‑1 (MQ=255)`  
                                                                                                                                                            `aacagCCTTTATGGACATGAAGATTCGATACACGATTCCTAGTGGAACATACAACGATGACACATTCACTACCTCTATTCATATTCCAACAATTTATGGTGCAAACTTCGGGAGGGGCAAAATCACTGTATTGGAGCCTGATAGTAAGATAACCGTGTTTGAGCAACCTACGGCTGGGTGGAATAGCGACCCTTGGCTGAGACTCAGCGGTAACTTGGAGGGACGCATGGTGTACATTGGGTTCAACATTAACTTCGTATATGAGTTCTCTAAGTTCCTCATCAAGCAGACTGCCgacgac                                                                                                                                               <  1:276790/297‑1 (MQ=255)`  
                                                                                                                                                                `accTTTATGGAAATGAAGATTCGATACAAGATTCCTAGTGGAACATACAACGATGACACATTCACTACCTCTATTCATATTCCAACAATTTATGGTGCAAACTTCGGGAGGGGCAAAATCAATGTATTGGAGCCTGATAGTAAGATAAACGTGTTTGGGCAAACTACGGATGGGTGGAATAGCGACCCTTGGCTGAGACTCAGCGGTAACTTGGAGGGACGAATGGTGTACATTGGGTTCAACATTAACTTCGTATATGAGTTCTCTAAGTTCCTCATCAAGCAGACTGCCGACGACGGGt                                                                                                                                           <  1:67957/300‑1 (MQ=255)`  
                                                                                                                                                                          `aCATGAAGATTCGATACACGATTCCTAGTGGAACATACAACGATGACACATTCACTACCTCTATTCATATTCAAACAATTTATGGTGCAAACTTCGGGAGGGGCAAAATCACTGTATTGGAGCCTGATAGTAAGATAACCGTGTTTGAGCAACCTACGGCTGGGTGGAATAGCGACCCTTGGCTGAGACTCAGCGGTAACTTGGAGGGACGCATGGTGTACATTGGGTTCAACATTAACTTCGTATATGAGTTCTCTAAGTTCCTCATCAAGCAGACTGCCGACGACGGGTCTACCTCCAc                                                                                                                                 <  1:139609/301‑1 (MQ=255)`  
                                                                                                                                                                           `cATGAAGATTCGATACACGATTCCTAGTGGAACATACAACGATGACACATTCACTACCTCTATTCATATTCCAACAATTTATGGTGCAAACTTCGGGAGGGGCAAAATCACTGTATTGGAGCCTGATAGTAAGATAACCGTGTTTGAGCAACCTACGGCTGGGTGGAATAGCGACCCTTGGCTGAGACTCAGCGGTAACTTGGAGGGACGCATGGTGTACATTGGGTTCAACATTAACTTCGTATATGAGTTCTCTAAGTTCCTCATCAAGCAGACCGACGACGACGGGTCTACCTACACg                                                                                                                                >  1:406330/1‑301 (MQ=255)`  
                                                                                                                                                                            `ggtgggctcggagatgtgtataagagacagaCATACAACTATTACACATTCAATACCTCTATTCATATTCCAAAAATTTATGGTGCAAACTTCGGGAGGGGCAAAATCACTGTATTGGAGCCTGATAGTAAGATAACCGTGGTTGAGCAACCTACGGCTGGGTGGAATAGCGACCCTTGGCTGAGACTCAGCGGTAACTTGGAGGGACGCATGGTGTACATTGGGTTCAACATTAACTTCGTATATGAGTTCTCTAAGTTCCTCATCAAGCAGACTGCCGACGACGGGTCTACCTCCACgg                                                                                                                               <  1:367958/271‑1 (MQ=255)`  
                                                                                                                                                                                  `aTTCGATACACGATTCCTAGTGGAACATACAACGATGACACATTCACTACCTCTATTCATATTCCAACAATTTATGGTGCAAACTTCGGGAGGGGCAAAATCACTGTATTGGAGCCTGATAGTAAGATAACCGTGTTTTAGCAACCTACGGCTGGGTGGAATAGCGACCCTTGGCTGAGACTCAGCGGTAACTTGGAGGGACGCATGGTGTACATTGGGTTCAACATTAACTTCGTATATGAGTTCTCTAAGTTCCTCATCAAGCAGACTGCCGACGACGGGTCTACCTCCACGGAAGAcc                                                                                                                         >  1:146135/1‑300 (MQ=255)`  
                                                                                                                                                                                   `ttCGATACACGATTCCTAGTGGAACATACAACGATGACACATTCACTACCTCTATTCATATTCCAACAATTTATGGTGCAAACTTCTGTAGGGGCAAAATCACTGTATTGGAGCCTGATAGTAAGATAACCGTGTTTGAGCAACCTACGGCTGGGTGGAATAGCGACCCTTGGCTGAGACTCAGCGGTAACTTGGAGGGACGCATGGTGTACATTGGGTTCAACATTAACTTCGTATATGAGTTCTCTAAGTTCCTCATCAAGCAGACTGCCGACGACGGGTCTACCTCCACGGAAGACAt                                                                                                                        <  1:132107/301‑1 (MQ=255)`  
                                                                                                                                                                                         `acacGATTCCTAGTGGAACATACAACGATGACACATTCACTACCTCTATTCATATTCCAACAATTTATGGTGCAAACTTCGAGAGGGGCAAAATCACTGTATTGGAGCCTGATAGTAAGATAACCGTGTTTGAGCAACCTACGGCTGGGTGGAATAGCGACCCTTTGCTGAGACTCAGCGGTAACTTGGAGGGACGCATGGTGTACATTGGGTTCAACATTAACTTCGTATATGAGTTCTCTAAGTTCCTCATCAAGCAGACTGCCGACGACGGGTCTACCTCCACGGAAGACATTGGgcg                                                                                                                  >  1:95968/1‑301 (MQ=255)`  
                                                                                                                                                                                              `aTTCCTAGTGGAACATACAACGATGACACATTCACTACCTCTATTCATATTCCAACAATTTATGGTGCAAACTTCGGGAGGGGCAAAATCACTGTATTGGAGCCTGATAGTAAGATAACCGTGTTTGAGCAACCTACGGATGGGTGGAATAGCGACCCTTGGCTGAGACTCAGCGGTAACTTGGAGGGACGCATGGTGTACATTGGGTTCAACATTAACTTCGTATATGAGTTCTCTAAGTTCCTCATCAAGCAGACTGCCGACGACGGGTCTACCTCCACGGAAGACATTGGGCGCTTAc                                                                                                             <  1:171505/301‑1 (MQ=255)`  
                                                                                                                                                                                                      `cggagatgtgtataagagacagttCACTACCTCTATTCATATTCCAACAATTTATGGTGCAAACTTCGGGAGGGGCAAAATCACTGTATTGGAGCCTGATAGTAAGATAACCGTGTTTGAGCAACCTACGGCTGGGTGGAATAGCGACCCTTGGCTGAGACTCAGCGGTAACTTGGAGGGACGCATGGTGTACATTGGGTTCAACATTAACTTCGTATATGAGTTCTCTAAGTTCCTCATCAAGCAGACTGCCGACGACGGGTCTACCTCCACGGAAGACATTGGGCGCTTACAGTTACGc                                                                                                     <  1:480252/279‑1 (MQ=255)`  
                                                                                                                                                                                                           `cATACAACGATGACACATTCACTACCTCTATTCATATTCCAACAATTTATGGTGCAAACTTCGGGAGGGGCAAAATCACTGTATTGGAGCCTGATAGTAAGATAACCGTGTTTGAGCAACCTACGGCTGGGTGGAATAGCGACCCTTGGCTGAGACTCAGCGGTAACTTGGAGGGACGCATGGTGTACATTGGGTTCAACATTAACTTCGTATATGAGTTCTCTAAGTTCCTCATCAAGCAGACTGCCGACGACGGGTCTACCTCCACGGAAGACATTGGGCGCTTACAGTTACGCCGAGc                                                                                                >  1:332717/1‑301 (MQ=255)`  
                                                                                                                                                                                                             `tACAACGATGACACATTCACTACCTCTATTCATATTCCAACAATTTATGGTGCAAACTTCGGGAGGGGCAAAATCACTGTATTGGAGCCTGATAGTAAGATAACCGTGTTTGAGCAACCTACGGCTGGGTGGAATAGCGACCCTTGGCTGAGACTCAGCGGTCACTTGGAGGGACGCATGGTGTACATTGGGTTCAACATTAACTTCGTATATGAGTTCTCTAAGTTCCTCATCAAGCATACTGCCGACGACGGGTCTACCTCCACGGAAGACATTGGGCGCTTACAGTTACGCCGAGCGt                                                                                              >  1:32872/1‑301 (MQ=255)`  
                                                                                                                                                                                                                  `cgttgtcacaTTCACTACCTCTATTCATATTCCAACAATTTATGGTGCAAACTTCGGGAGGTGCAAAATCACTGTATTGGAGCCTGATAGTAAGATAACCGTGTTTGAGCAACCTACGGCTGGGTGGAATAGCGACCCTTGGCTGAGACTCAGCGGTAACTTGGAGGGACGCATGGTGTACATTGGGTTCAACATTAACTTCGTATATGAGTTCTCTAAGTTCCTCATCAAGCAGACTGCCGACGACGGGTCTACCTCCACGGAAGACATTGGGCGCTTACAGTTACGCCGAGCGTGGGtt                                                                                         <  1:63752/295‑1 (MQ=255)`  
                                                                                                                                                                                                                  `cGATGACACATTCACTACCTCTATTCATATTCCAACAATTTATGGTGCAAACTTCGGGAGGGGCAAAATCACTGTATTGGAGCCTGATAGTAAGATAACCGTGTTTGAGCAACCTACGGCTGGGTGGAATAGCGACCCTTGGCTGAGACTCAGCGGTAACTTGGAGGGACGCATGGTGTACATTGGGTTCAACATTAACTTCGTATATGAGTTCTCTAAGTTCCTCATCAAGCAGACTGCCGACGACGGGTCTACCTCCACGGAAGACATTGGGCGCTTACAGTTACGCCGAGCGTGGGtt                                                                                         >  1:21298/1‑301 (MQ=255)`  
                                                                                                                                                                                                                       `aagaccgcattACCTCTATTCAAATTCCAACAATTTATGGTGCAAACTTCGGGAGGGGCAAAATCACTGTATTGGAGCCTGATAGTAAGATAACCGTGTTTGAGCAACCTACGGGTGGGTGGAATAGCGACCCTTGGCTGAGACTCAGCGGTAACTTGGAGGGACGCATGGTGTACATTGGATTCAACATTAACTTCGTATATGAGTTCTCTAAGTTCCTCATCAAGCAGGCTGCCGACGACGGGTCTACCTCCACGGAAGACATTGGGCGCTTACAGTTACGCCGAGCGTGGGTTAACTa                                                                                    <  1:195158/291‑1 (MQ=255)`  
                                                                                                                                                                                                                            `ttCACTACCTCTATTCATATTCCAACAATTTATGGTGCAAACTTCGGGAGGGGCAAAATCACTGTATTGGAGCCTGATAGTCAGATAACCGTGTTTGCGCAACCTACGGCTGGGTGGAATAGCGACCCTTGGCTGAGACTCAGCGGTAACTTTACGGGACGCATGGTGTACATTGGGTTCAACATTACCTACATATATGAGTTATCTAAGTTCCTCATAAAGCAGACTGCCGACGACGGGTCTACCTCCACGGAAGCCATTGGCCGCTTCCAGTTACGCCGAGCGTGTGTTACCTCCgaga                                                                               >  1:489894/1‑301 (MQ=255)`  
                                                                                                                                                                                                                            `ttCACTACCTCTATTCATATTCCAACAATTTATGGTGCAAACTTCGGGAGGGGCAAAATCACTGTATTGGAGCCTGATAGTAAGATAACCGTGTTTGAGCAACCTACGGCTGGGTGGAATAGCGACCCTTGGCTGAGACTCATCGGTAACTTGGAGGGACGCATGGTGTACATTGGGTTCAACATTAACTTCGTATATGAGTTCTCTAAGTTCCTCATCAAGCAGACTGCCGACGACGGGTCTACCTCCACGGAAGACATTGGGCGCTTACAGTTACGCCGAGCGTGGGTTAACTACgaga                                                                               >  1:344559/1‑301 (MQ=255)`  
                                                                                                                                                                                                                             `tCACTACCTCTATTCATATTCCAACAATTTATGGTGCAAACTTCGGGAGGGGCAAAATCACTGTATTGGAGCCTGATAGTAAGATAACCGTGTTTGAGCAACCTACGGCTGGGTGGAATAGCGACCCTTGGCTGAGACTCAGCGGTAACTTGGAGGGACGCATGGTGTACATTGGGTTCAACATTAACTTCGTATATGAGTTCTCTAAGTTCCTCATCAAGCAGACTGCCGACGACGGGTCTACCTCCACGGAAGACATTGGGCGCTTACAGTTACGCCGAGCGTGGGTTAACTACGAGaa                                                                              <  1:123798/301‑1 (MQ=255)`  
                                                                                                                                                                                                                             `gcACTACCTCTATTCATATTCCAACAATTTATGGTGCAAACTTCGGGAGGGGCAAAATCACTGTATTGGGGCCTGATAGTAAGATAACCGTGTTTGAGCAACCTACGGCTGGGTGGAATAGCGACCCTTGGCTGAGACTCAGCGGTAACTTGGAGGGACGCATGGTGTACATTGGGTTCAACATTAACTTCGTATATGAGTTCTCTAAGTTCCTCATCAAGCAGACTGCCGACGACGGGTCTACCTCCACGGAAGACATTGTGCGCTTACAGTTACGCCGAGCGTGGGTTAACTACGAGaa                                                                              <  1:458699/300‑1 (MQ=255)`  
                                                                                                                                                                                                                              `cACTACCTCTATTCATATTCCAACAATTTATGGTGCAAACTTCGGGAGGGGCAAAATCACTGTATTGGAGCCTGATAGTAAGATAACCGTGTTTGAGCAACCTACGGCTGGGTGGAATAGCGACCCTTGGCTGAGACTCAGCGGTAACTTGGAGGGACGCATGGTGTACATTGGGTTCAACATTAACTTCGTATATGAGTTCTCTAAGTTCCTCATCAAGCAGACTGCCGACGACGGGTCTACCTCCACGGAAGACATTGGGCGCTTACAGTTACGCCGAGCGTGGGTTAACTCCGAGAAc                                                                             >  1:381150/1‑301 (MQ=255)`  
                                                                                                                                                                                                                              `cACTACCTCTATTCATATTCCAACAATTTATGGGGCAAACTTCGGGAGGGGCAAAATCACTGTATTGGAGCCTGGTAGTAAGATAACCGTGTTTTAGCAACCTACGGCTGGGTGGAATAGCGACCCTTGTCTGAGACTCAGCGGTAACTTGGAGGGACGCATGGTGTACATTGGGTTCAACATTAACTTCGTATATGAGTTCTCTAAGTTCCTCATCAAGCAGACTGCCGACGACGGGTCTACCTCCACGGAAGACATTGGGCGCTTACAGTTACGCCGAGCGTGGGTTAACTACGAGAAc                                                                             <  1:86955/301‑1 (MQ=255)`  
                                                                                                                                                                                                                              `aaCTACCTCTATTCATATTCCAACAATTTAAGGTGCAAACTTCGGGAGGGGCAAAATCACTGTAATGGAGCCTGATAGTAAGATAACCGTGTTTGAGCAACCTACGGCTGGGTGGAATAGCGACCCTTGGCTGAGACTCAGCGGTAACTTGGAGCGACGCATGGTGTACATTGGGTTCAACATTAACTTCGTATATGAGTTCTCTAAGTTCCTCATCAAGCAGACTGCCGACGACGGGTCTACCTCCACGGAAGACATTGTGCGCTTACAGTTACGCCGAGCGTGGGTTAACTACGAGAAc                                                                             <  1:184405/300‑1 (MQ=255)`  
                                                                                                                                                                                                                                   `ccTCTATTCATATTCCAACAATTTATGGTGCAAACTTCGGGAGGGGCAAAATCACTGTATTGGAGCCTGATAGTAAGATAACCGTGTTTGAGCAACCTACGGCTGGGTGGAATAGCGACCCTTGGCTGAGACTCAGCGGTAACTTGGAGGGACGCATGGTGTACATTGGGTTCAACATTAACTTCGTATATGAGTTCTCTAAGTTCCTCATCAAGCAGACTGCCGACGACGGGTCTACCTCCACGGAAGACATTGGGCGCTTACAGTTACGCCGAGCGTGGGTTAACTACGAGAACTCTgg                                                                        <  1:189056/301‑1 (MQ=255)`  
                                                                                                                                                                                                                                      `ctATTCATATTCCAACAATTTATGGTTCAAACTTCGGGAGGGGCAAAATCACTGTATTGGAGCCTGATAGGAAGATAACCGTGTTTGAGCAACCTACGGCTGGGTGGAATAGCGACCCTTGGCTGAGACTCAGCGGTAACGTGGAGGGACGCATGGTGTACATTGGGTTCAACATTAACTTCGTATATGAGTTCTCTAAGTTCCTCATCAAGCAGACTGCCGACGACGGGTCTACCTCCACGGAAGACATTGGGCGCTTACAGTTACGCCGAGCGTGGGTTAACTACGAGAACTCTGGTAc                                                                     <  1:120167/301‑1 (MQ=255)`  
                                                                                                                                                                                                                                        `gtgggctcggagatgtgtataagagacagcTTCGGGAGGGGCAAAATCACTGTATTGGAGCCTGATAGTAAGATAACCGTGTTTGAGCAACCTACGGCTGGGTGGAATAGCGACCCTTGGCTGAGACCCAGCGGTAACTTGGAGGGACGCATGGTGTACATTGGGTTCAACATTAACTTCGTATATGAGTTCTCTAAGTTCCTCATCAAGTAGACTGCCGACGACGGGTCTACCTCCACGGAAGACATTGGGCGCTTACAGTTACGCCGAGCGTGGGTTAACTACGAGAACTCTGGTACGt                                                                   <  1:397253/272‑1 (MQ=255)`  
                                                                                                                                                                                                                                        `aTTCATATACCAACAATTTATGGTGCAAACTTCGGGAGGGGCAAAATCACTGTATTGGAGCCTGATAGTAAGATAACCGTGTTTGAGCAACCTACGGCTGGGTGGAATAGCGACCCTTGGCTGAGACTCAGCGGTAACTTGGAGGGACGCATGGTGTACATTGGGTTCAACATTAACTTCGTATATGAGTTCTATAAGTTCCTCATCAAGCAGACTGCCGACGACGGGTCTACCTTCACGGAAGACATTGGGCGCTTACAGTTACGCCGAGCGTGGGTTAACTACGAGAACTCTGGTACGt                                                                   >  1:216793/1‑301 (MQ=255)`  
                                                                                                                                                                                                                                                     `aaaTTTATGGTGCAAACTTCGGGAGGGGCAAAATCACTGTATTGGAGCCTGATAGTAAGATAACCGTGTTTGAGCAACCTACGGCTGGGTGGAATAGCGACCCTTGGCTGAGACTCAGCGGTAACTTGGAGGGACGCATGGTGTACATTGGGTTCAACATTAACTTCGTATATGAGTTCTCTAAGTTCCTCATCAAGCAGACTGCCGACGACGGGTCTACCTCCACGGAAGACATTGGGCGCTTACAGTTACGCCGAGCGTGGGTTAACTACGAGAACTCTGGTACGTTTGACATTTATGt                                                      <  1:409651/300‑1 (MQ=255)`  
                                                                                                                                                                                                                                                                  `aaaCTTCGGGAGGGGCAAAATCACTGTATTGGAGCCTGATAGTAAGATAACCGTGTTTGAGCAACCTACGGCTGGGTGGAATAGCGACCCTTGGCTGAGACTCAGCGGTAACTTGGAGGGACGCATGGTGTACATTGGGTTCAACATTAACTTCGTATATGAGTTCTCTAAGTTACTCATCAAGCAGACTGCCGACGACGGGTCTACCTCCACGGAAGACATTGGGCGCTTACAGTTACGCCGAGCGTGGGTTAACTACGAGAACTCTGGTACGTTTGACATTTATGTTGAGAACCAATCg                                         <  1:77179/301‑1 (MQ=255)`  
                                                                                                                                                                                                                                                                        `cGGGAGGGGCAAAATCACTGTATTGGAGCCTGATAGTAAGATAACCGTGTTTTAGCAACCTACGGCTGGGTGGAATAGCGACCCTTGGCTGAGACTCAGCGGTAACTTGGAGGGACGCATGGTGTACATTGGGTTCAACATTAACTTCGTATATGAGTTCTCTAAGTTCCTCATCAAGCAGACTGCCGACGACGGGTCTACCTCCACGGAAGACATTGGGCGCTTACAGTTACGCCGAGCGTGGGTTAACTACGAGAACTCTGGTACGTTTGACATTTATGTTGAGAACCAATCGTCTAAc                                   <  1:401377/301‑1 (MQ=255)`  
                                                                                                                                                                                                                                                                              `gggtatattcgtggTATTGGAGCCGAGTAGTAAGATTACCTTGTTTGATCAACCTACGGCTGGGTGGAATAGCGACCCTTGTCTGAGTCTCAGCGGTAACTTGGAGTGACGCATGGTGTACAGTGGGTGCAACATTAACTTAGTATATGATGTCTCTAAGTTCCTCATCAAGCAGACTGCCGACGACGGGTCTACCTCCACGGAAGACATTGGGCGCTTACAGATACGCCTGGCGTGGGTTAACTACGAGAACTCTGGTACGTTTGACATTTATGTTGAGAACCAATCGTCTAACTGGAAg                             <  1:61143/288‑1 (MQ=255)`  
                                                                                                                                                                                                                                                                               `ggCAAAATCACTGTATTGGAGCCTGATAGTAAGATAACCGTGTTTGAGCAACCTACGGCTTGGTGGAATAGCGACCCTTGGCTGAGACTCAGCGGTAACTTGGAGGGACGCATGGTTTACATTGGGTTCAACATTAACTTCGTATATGCGTTCTCTACGTTACTCATCAAGCAGACTGCCGACGACGGGTCTACCTCCACGGAAGACATTGGGCGCTTACAGTTACTCCGAGAGTGGGTTAACTACGAGAACTCTGGTACGTTTGACATTTATGTTGAGAACCAATCGTCTAACTTGaact                            >  1:386674/1‑299 (MQ=255)`  
                                                                                                                                                                                                                                                                                   `aaaTCACTGTATTGGGTGCTGATAGTAAGATAACCGTGTTTGAGCAACCTACGGCTGGGTGGAATAGCGACCCTTGGCTGAGACTCAGCGGTAACTTGGAGGGACGCATGGTGTACATTGGGTTCAACATTAACTTCGTATATGAGTTCTCTAAGTTCCTCATCAAGCAGACTGCCGACGATGGGTCTACCTCCACGGAAGACATTGGGCGCTTACAGTTACGCCGAGCGTGGGTTAACTACGAGAACTCTGGTACGTTTGACATTTATGTTGAGAACCAATCGTCTAACTGGAAGTacac                        <  1:418155/301‑1 (MQ=255)`  
                                                                                                                                                                                                                                                                                   `aaaTCACTGTATTGGAGCCTGATAGTAAGATAACCGTGTTTGAGCAACCTACGGCTGGGTGGGATAGCGACCCTTGGCTGAGACTCAGCGGTAACTTGGAGGGACGCATGGTGTACATTGGGTTCAACATTAACTTCGTATATGAGTTCTCTAAGTTCCTCATCAAGCAGACTGCCGACGACGGGTCTACCTCCACGGAAGACATTGGGCGCTTACAGTTACGCCGAGCGTGGGTTAACTACGAGAACTCTGGTACGTTTGACATTTATGTTGAGAACCAATCGTCTAACTGGAAGTacac                        <  1:261985/301‑1 (MQ=255)`  
                                                                                                                                                                                                                                                                                   `aaaTCACTGTATTGGAGCCTGATAGTAAGATAACCGTGTTTGAGCAACCTACGGCTGGGTGGAATAGCGACCCTTGGCTGAGACTCAGCGGTAACTTGGAGGGACGCATGGTGTACATTGGGTTCAACATTAACTTCGTATATGAGTTCTCTAAGTTCCTCATCAAGCAGACTGCCGACGACGGGTCTACCTCCACGGAAGACATTGGGCGCTTACAGTTACGCCGAGCGTGGGTTAACTACGAGAACTCTGGTACGTTTGACATTTATGTTGAGAACCAATCGTCTAACTGGAAGTacac                        <  1:413901/301‑1 (MQ=255)`  
                                                                                                                                                                                                                                                                                   `aaaTCACTGTATTGGAGCCTGATAGTAAGATAACCGTGTTTGAGCAACCTACGGCTGGGTGGAATAGCGACCCTTGGCTGAGACTCAGCGGTAACTTGGAGGGACGCATGGTGTACATTGGGTTCAACATTAACTTCGTATATGAGTTCTATAAGTTCCTCATCAAGCAGACTGCCGACGACGGGTCTACCTCCACGGAAGACATTGGGCGCTTACAGTTACGCCGAGCGTGGGTTAACTACGAGAACTCTGGTACGTTTGACATTTATGTTGAGAACCAATCGTCTAACTGGAAGTacac                        <  1:362541/301‑1 (MQ=255)`  
                                                                                                                                                                                                                                                                                     `aTCACTGTATTGGAGCCTGATAGTAAGATAACCGTGTTTGAGCAACCTACGGCTGGGTGGAATAGCGACCCTTGGCTGAGACTCAGCGGTAACTTGGAGGGACGCATGATGTACATTGGGTTCAACATTAACTTCGTATATGAGTTCTCTCAGTTCCTCATAAAGCAGACTGACGACGACGGGTCTAACTGCTCCGACGACACTGGGTGCTTACAGTTACGCCGAGCGTGGGTTAACTCAGAGCAATCTGGTACGTTTGACATTTATGTTGAGAACCAATCGTCTAACTGGagttccctgt                      >  1:439097/1‑292 (MQ=255)`  
                                                                                                                                                                                                                                                                                      `tCACTGTATTGGAGCCTGATAGTAAGATAACCGTGTTTGAGCAACCTACGGCTGGGTGGAATAGCGACCCTTGGCTGAGACTCAGCGGTAACTTGGAGGGACGCATGGTGTACATTGGGTTCAACATTAACTTCGTATATGAGTTCTCTAAGTTCCTCATCAAGCAGACTGCCGACGACGGGTCTACCTCCACGGAAGACATTGGGCGCTTACAGTTACGCCGAGCGTGGGTTAACTACGAGAACTCTGGTACGTTTGACATTTATGTTGCGAAGCAATCGTCTAACTGGAAGTACACAAt                     >  1:339170/1‑301 (MQ=255)`  
                                                                                                                                                                                                                                                                                      `tCACTGTATTGGAGCCTGATAGTAAGATAACCGTGTTTGAGCAACCTACGGCTGGGTGGAATAGCGACCCTTGGCTGAGACTCAGCGGTAACTTGGAGGGACGCATGGTGTACATTGGGTTCAACATTAACTTCGTATATGAGTTCTCTAAGTTCCTCATCAAGCAGACTGCCGACGACGGGTCTACCTCCACGGAAGACATTGGGCGCTTACAGTTACGCCGAGCGTGGGTTAACTACGAGAACTCTGGTACGTTTGACATTTATGTTGAGAACCAATCGTCTAACTGGAAGTACACAAt                     >  1:157750/1‑301 (MQ=255)`  
                                                                                                                                                                                                                                                                                      `tCACTGTAGTGGAGCCTGATAGTAAGATAACCGTGTTTGAGCAACCTACGGCTGGGTGGAATAGCGACCCTTGGCTGAGACTCAGCGGTAACTTGGAGGGACGCATGGTGTACATTGGGTTCAACATTAACTTCGTATATGAGTTCTCTAAGTTCCTCATCAAGCAGACTGCCGACGACTGGTCTACCTCCACGGAAGACATTGGGCGCTTACAGTTACGCCGAGCGTGGGTTAACTACGAGAACTCTGGTACGTTTGACATTTATGTTGAGAACCAATCGTCTAACTGGAAGTACACAAt                     <  1:271813/301‑1 (MQ=255)`  
                                                                                                                                                                                                                                                                                          `tGTATTGGAGCCTGATAGTAAGATAACCGTGTTTGAGCAACCTACGGCTGGGTGGAATAGCGACCCTTGGCTGAGACTCAGCGGTAACTTGGAGGGACGCATGGTGTACATTGGGTTCAACATTAACTTCGTATATGAGTTCTCTAAGTTCCTCATCAAGCAGACTGCCGACGACGGGTCTACCTCCACGGAAGACATTGGGCGCTTACAGTTACGCCGAGCGTGGGTTAACTACGAGAACTCTGGTACGTTTGACATTTATGTTGAGAACCAATCGTCTAACTGGAAGTACACAATGGCt                 <  1:152769/301‑1 (MQ=255)`  
                                                                                                                                                                                                                                                                                           `gTATTGGAGCCTGATAGTAAGATAACCGTGTTTGAGCAACCTACGGCTGGGTGGAATAGCGACCCTTGGCTGAGACTCAGCGGTAACTTGGAGGGACACATGGTGTACATTGGGTTCAACATTAACTTCGTATATGAGTTCTCTAAGTTCCTCATCAAGCAGACTACCGACGACGGGTCTACCTCCACGGAAGACATTGGGCGCTTACAGTTACGCCGAGCGTGGGTTAACTACGAGCACTCTGGTACGTTTGACATTTATGTTGAGAACCAATCGTCTAACTGGAAGTACACAATGGCTg                >  1:266884/1‑301 (MQ=255)`  
                                                                                                                                                                                                                                                                                            `taatGGAGCCTGATAGTAAGATAACCGTGTTTTAGCAACCTACGGCTGGGTGGAATAGCGACCCTTGGCTGAGACTCAGCGGTAACTTGGAGGGACGCATGGTGTACATTGGGTTCAACATTAACTTCGTATATGAGTTCTCTAAGTTCCTCATCAAGCAGACTGCCGACGACGGGTCTACCTCCACGGAAGACATTGGGCGCTTACAGTTACGCCGAGCGTGGGTTAACTACGAGAACTCTGGTACGTTTGACATTTATGTTGAGAACCAATCGTCTAACTGGAAGTACACAATGGCTgg               <  1:175896/298‑1 (MQ=255)`  
                                                                                                                                                                                                                                                                                                    `ccTGATAGTAAGATAACCGTGTTTGAGCAACCTACGGCTGGGTGGAATAGCGACCCTTGGCTGAGACTCAGCGGTAACTTGGAGGGACGCATGGTGTACATTGGGTTAAACATTAACTTCGTATATGAGTTCTCTAAGTTCCTCATCAAGCAGACTGCCGACGACGGGTCTACCTCCACGGAAGACATTGGGCGCTTACAGTTACGCCGAGCGTGGGTTAACTACGAGAACTCTGGTACGTTTGACATTTATGTTGAGAACCAATCGTCTAACTGGAAGTACACAATGGCTGGTGCCCGAt       >  1:72015/1‑301 (MQ=255)`  
                                                                                                                                                                                                                                                                                                       `gATAGTAAGATAACCGTGTTTGAGCAACCTACGGCTGGGTGGAATAGCGACCCTTGGCTGAGACTCAGCGGTAACTTGGAGGGACGCATGGTGTACATTGGGTTCAACATTAACTTCGTATATGAGTTCTCTAAGTTCCTCATCAAGCAGACTGCCGACGACGGGTCTACCTCCACGGAAGACATTGGGCGCTTACAGTTACGCCGATCGTGGGTTAACTACGAGAACTCTGGTACGTTTGACATTTATGTTGAGAACCAATCGTCTAACTGGAAGTACACAATGGCTGGTGCCCGATTAg    >  1:11608/1‑301 (MQ=255)`  

                                                                                                                                                                                                                                                                                                          `|`                                                                                                                                                                                                                                                                                                             
`GTCGTGGTCTCATTGGGACTTTGGGGAAAACGTACAGGTTCTAGCTTGTCAGAGTATCAGCTCAGATATGTATGTGATTCTTCGCAATGAGTTCAATACGTTCCTAGCTAGAATCTCTTTCACTAAGAACGCCATTGACTTACAGGGAGAACCCTATCGTGCCTTTATGGACATGAAGATTCGATACACGATTCCTAGTGGAACATACAACGATGACACATTCACTACCTCTATTCATATTCCAACAATTTATGGTGCAAACTTCGGGAGGGGCAAAATCACTGTATTGGAGCCTGATGGTAAGATAACCGTGTTTGAGCAACCTACGGCTGGGTGGAATAGCGACCCTTGGCTGAGACTCAGCGGTAACTTGGAGGGACGCATGGTGTACATTGGGTTCAACATTAACTTCGTATATGAGTTCTCTAAGTTCCTCATCAAGCAGACTGCCGACGACGGGTCTACCTCCACGGAAGACATTGGGCGCTTACAGTTACGCCGAGCGTGGGTTAACTACGAGAACTCTGGTACGTTTGACATTTATGTTGAGAACCAATCGTCTAACTGGAAGTACACAATGGCTGGTGCCCGATTAGGC  >  NC_001604.1/26455‑27052`

  

Alignment Legend

Aligned base mismatch/match (shaded by quality score): `ATCG/ATCG` `< 3 ≤` `ATCG/ATCG` `< 10 ≤` `ATCG/ATCG` `< 20 ≤` `ATCG/ATCG` `< 34 ≤` `ATCG/ATCG` `< 38 ≤` `ATCG/ATCG`

Unaligned base: `atcg`    Masked matching base: `atcg`    Alignment gap: `‑`    Deleted base: `‑`
